# Normal Baseline Values for Isometric Shoulder Strength in Scaption for Healthy Filipino Individuals in the 20-30 year Age Group

**DOI:** 10.5704/MOJ.2107.007

**Published:** 2021-07

**Authors:** PH Lorenzo, R Nunez

**Affiliations:** Department of Orthopaedic Surgery, University of Santo Tomas Hospital, Manila, Philippines

**Keywords:** isometric shoulder strength, hand-held dynamometer, normative database

## Abstract

**Introduction:**

Isometric shoulder strength is vital in the management of individuals suffering from shoulder diseases such as rotator cuff tears. Normal values for the working Filipino population who are at risk of developing shoulder problems are lacking. The objective of this study was to determine the isometric baseline isometric shoulder strengths in scaption of healthy Filipino individuals aged 20-30 years old without a history of a shoulder injury.

**Material and Methods:**

This is a cross-sectional descriptive study measuring the isometric strength values using the handheld IDO isometer of dominant and non-dominant shoulder of healthy Filipino individuals aged 20 to 30 years old.

**Results:**

There is no significant difference in the mean isometric shoulder strength between the dominant and non-dominant arm for both sexes. The male gender scored higher values compared to the female gender and is statistically significant.

**Conclusion:**

There is no difference in isometric shoulder strength between the dominant and non-dominant shoulder. Strength differences favour the male gender.

## Introduction

In clinical practice, a variety of conditions affect the upper limb strength^1^. Measurement of shoulder strength plays a vital role in the management of the patient in terms of diagnosis, therapeutic intervention, and evaluation of treatment outcomes^1-4^. Strength testing is performed to assess the ability of the patient to maintain a degree of tension in the voluntary muscle against a force or resistance^5^. It can be tested using the traditional manual muscle grading and assigning a grade from 0 to 53,5. However, such assessment lacks interrater reliability and is limited since it cannot be expressed in standard units^5^.

Several methods to objectively measure shoulder strength have been made available including the use of isometric dynamometer, cable tensiometer, and spring balance. In addition to giving an objective measure of strength these methods of measuring strength are particularly applied in a clinical research setting as components of standardised functional assessment tools such as the Constant-Murley Score^5^. The Constant- Murley Score is a 100-point scale with components measuring the functionality of the shoulder. Of the 100 points, 25 points are allotted to assess the strength of the shoulder^5^. The tested shoulder is positioned in 90° of abduction elbow expended and forearm pronated with 30° of horizontal adduction which is termed as the angle of scaption. This is the position where the arm is positioned in the plane of the scapula. Elevation or flexion of the arm in the plane of the scapula is a functional movement utilised in everyday life^6^. Although vital in everyday life, little is known about the expected normal baseline of shoulder strength in this position.

The primary objective was to determine the isometric baseline isometric shoulder strength in scaption of healthy Filipino individuals aged 20-30 years old without a history of a shoulder injury. Secondary objectives were to compare the isometric shoulder strength values between the male and female gender and to compare the isometric shoulder strength values between the dominant and non-dominant arm.

## Materials and Methods

This is a descriptive cross-sectional study with recruitment of participants and gathering of data was done by the investigator on the same day once inclusion criteria are fulfilled and consent given by the participant. The study was approved by the Institutional Research Ethics Committee of the University of Santo Tomas Hospital (REC-2019-04-086-IS).

Sample size computation using post-hoc power analysis for two sample means (independent t-test) was conducted using GPower [version 3.1.7.] showed at least a minimum of 156 respondents to achieve a power of 80% and an effect size of 0.453 at a significance level of 5% (two-tailed). The sample size was further divided into two groups – 78 males and 78 females. All eligible healthy Filipino individuals aged 20 to 30 years old without a history of shoulder trauma were included. Exclusion criteria were: (1) individuals with shoulder symptoms (pain, instability, limitation of motion), (2) Individuals with a history of previous shoulder trauma and or shoulder surgery, and (3) Individuals with a history of neurological disease.

**Fig. 1:**
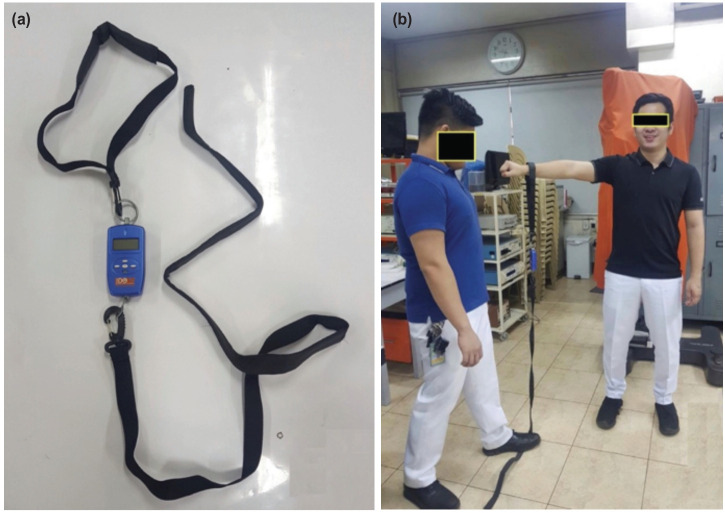
(a) Handheld IDO isometer, (b) test position for participants.

A baseline questionnaire for demographics was filled up by the participants. A brief physical examination by the investigator was done to assess the range of motion, tenderness, and joint instability. Bilateral shoulder strength testing was done using the handheld IDO Isometer (Fig. 1a) participants were positioned in the standing position. Shoulder positioned in 90° abduction with 30° horizontal adduction. The strap was placed in the wrist over the caput ulnae with the assessor in-line with the upper extremity stepping on the other end of the strap of the isometer (Fig. 1b). Once positioned, participants were asked to push maximally upward for 3 seconds. Verbal cue and encouragement: “Ready 3, 2, 1, push push push!” was done for the duration of the testing. A one trial warm-up was done before the actual testing for each shoulder. Three measurements for each arm were done starting with the dominant arm followed by the non-dominant arm. The highest value among the three measurements was recorded for statistical analysis.

The outcome of interest of the study is the digital value registered in the IDO Isometer in kilograms for both the dominant and non-dominant arm. The statistical analyses were performed using Microsoft Excel. A p-value ≤0.05 is considered significant. Descriptive statistics included mean and standard deviation. Comparative analysis between the dominant and non-dominant hands was estimated using a paired t-test, while comparison between sexes was conducted using an independent t-test.

## Results

A total of 156 participants were included in the study. Seventy-eight (78) males and 78 females with a mean age of 24±1 and 24±0.8, respectively. The mean isometric strength of the shoulder in scaption for the dominant and non-dominant arm is shown in (Table I). There is no significant difference between the shoulder strength of dominant and non-dominant arm for both males and females (male: p value=0.97; female: p-value = 0.16). However, the male gender compared to the female gender shows a significant difference in isometric shoulder strength (p-value = 0.002) male showing stronger isometric strength.

**Table I:** Mean Isometric Shoulder strength in kilograms (kg) of male and female participants

	Male (n=78) kg	Female (n=78) kg
Dominant Arm	1.61 ± 0.83	0.89 ± 0.39
Non-dominant Arm	1.61 ± 0.78	0.85 ± 0.38

## Discussion

Isometric strength testing using a handheld dynamometer is an efficient and inexpensive way for clinicians to measure the strength of a muscle group^2,5,7,8^. Available instruments to measure the isometric strength has been proved to be reliable and show reproducible results^5,7,9^. Results of isometric strength testing are comparable to isokinetic strength testing which is the gold standard^2^.

Isometric shoulder strength has been reported across different age groups and in different athletes^2,8,10,11^. Westrick *et al* measured the isometric strength of active collegiate males and females aged 17 to 21 years old in abduction, flexion, internal and external rotation results of the study showed that for both genders the dominant shoulder is being significantly stronger compared to the non-dominant shoulder also males were significantly stronger than females^2^. One limitation of the was a relatively dominant male population. Cools *et al* measured the eccentric and isometric shoulder strengths for internal and external rotation of 201 overhead athletes aged 18 to 50 years old with equal male and female participants results showed that strength differences favour the dominant side and the male gender^8^. However, when normalised to body weight gender differences are absent^8^. Also, the isometric strength values of handball and tennis players were stronger compared to volleyball players owing to possible sport-specific adaptations^8^. McLaine *et al* measured the isometric strength of male and female swimmers aged 14 to 20 years old results showed higher values for the male gender. However, in contrast to other studies, no difference was found between the dominant and non-dominant shoulders owing to the bilateral nature of the sport justifying sport-specific adaptations^10^.

Kim *et al* reported the normal baseline isometric strength of shoulder in scaption which was done in healthy individuals aged 40 years old and above showing that age is the most important predictor of strength in abduction^11^. Also, the same study determined the effect of asymptomatic rotator cuff tears in shoulder abduction strength in scaption the study showed that a substantial decrease in strength of shoulder abduction in scaption in relation to strength in external rotation a possibility of an asymptomatic rotator cuff tear should be suspected^11^. A study done by Yamaguchi *et al* concluded that there is a 56.3% chance of rotator cuff tear in the contralateral asymptomatic shoulder if a patient presents with a painful full-thickness tear in one shoulder^12^.

Balcells-Diaz *et al* with the objective of normalisation of Constant score in an epidemiological study reported the variation in baseline strength measurements across different age groups and genders. The population of the study was adult individuals aged 18 years and above. The study concluded that male gender is stronger compared to the female counterparts and a decline of shoulder strength occurs with an increase in age^13^.

The results in this study showed no significant difference of isometric shoulder strength between the dominant and non-dominant arm for both sexes which is consistent with the previous studies reported. Also, similar to previous studies the male gender has stronger shoulder strength compared to the female counterparts. Our study can also be of value to individuals undergoing physical therapy as this can serve as reference values to aid their rehabilitation prior to returning to normal activity. Limitations of the study are the non-adjustment of body weight for differences in sexes which was done by Cools *et al*^8^ Although the sample size was fulfilled a larger sample size not just to increase the power if but also to cover a wider age range to further determine the baseline strength across different age groups is further recommended by the authors.

## Conclusion

In the evaluation of shoulder function in patients with bilateral rotator cuff tear neither shoulder can be used as a reliable reference for strength because a tear can alter the biomechanics of the shoulder. Thus, to achieve an accurate objective evaluation a normative database of healthy subjects with intact rotator cuff should be established. To the authors' knowledge, this is the first normative data study in the country looking at isometric shoulder strengths in this age group. There is no difference in strength between the dominant and non-dominant arm for both male and female genders; however, the strength difference favours the male gender.
